# Neuroendocrine liver metastasis from the small intestine: Is surgery beneficial for survival?

**DOI:** 10.1186/s13023-021-01677-9

**Published:** 2021-01-14

**Authors:** Andreas Selberherr, Simon Freermann, Oskar Koperek, Martin B. Niederle, Philipp Riss, Christian Scheuba, Bruno Niederle

**Affiliations:** 1 Section “Endocrine Surgery”, Division of General Surgery, Department of Surgery, Medical University, Währinger Gürtel 18-20, 1090 Vienna, Austria; 2 Department of Pathology, Medical University, Währinger Gürtel 18-20, 1090 Vienna, Austria; 3 Department of Anesthesiology, Medical University, Währinger Gürtel 18-20, 1090 Vienna, Austria

**Keywords:** Neuroendocrine tumors, Neuroendocrine neoplasia, Livermetastasis, NET, NEN, Small intestine

## Abstract

**Background:**

Neuroendocrine neoplasia of the small intestine (siNEN) are frequently diagnosed with liver metastases. The impact of the presence of liver metastases on overall survival and the necessity of surgery for liver metastasis is discussed controversially. The aim of this study is to evaluate and compare the overall long-term survival of patients with siNENs with and without liver metastasis at initial diagnosis and the possible benefit of surgical treatment as compared to active surveillance of metastases. 123 consecutive patients with siNENs were treated between 1965 and 2016. All clinical and histological records were reevaluated including analysis of the proliferation rates in all specimens. The 1-, 5-, 10- and 20-year overall survival was estimated by Kaplan–Meier analysis for patients with and without liver metastasis and according to the type of treatment (surgical vs. surveillance) of liver metastases if present.

**Results:**

The 1-, 5-, 10- and 20-year overall survival rate was 89.0%, 68.4%, 52.8% and 31.0% in patients without and 89.5%, 69.5%, 33.2% and 3.6% in those with liver metastases. No statistically significant differences were observed comparing the two groups. Within the group of patients with liver metastases, the type of treatment (surgical vs. surveillance) was in favor of patients undergoing surgery. Multivariate analysis showed that the presence of liver metastases upon diagnosis was an individual risk factor associated with worse survival.

**Conclusion:**

The presence of liver metastasis at initial diagnosis does not have a statistically significant influence on survival. Surgery for hepatic metastasis seems to show a benefit for overall survival and may be indicated especially in patients symptomatic due to high tumor burden and serotonin hypersecretion to reduce hormone activity.

## Background

Neuroendocrine neoplasia (NEN) of the small intestine (si) are rare tumors with an estimated incidence of 0.29 per 100,000 [1, 2]. SiNENs remain asymptomatic for a long time because of the late onset of symptoms which are most commonly unspecific. Elevated levels of serotonin (5-HT) which correlate with tumor burden may cause carcinoid syndrome that is characterized by diarrhea, flushing, sweating and palpitations [3, 4]. Diagnosis is frequently made in late stage with/without serotonin related symptoms or due to bowel obstruction caused by the tumor mass [5]. In about 36% of patients, metastases are already present at initial diagnosis [6].

In the early stages of disease (I–III A (= N0, M0)] the treatment of choice is radical surgery of the primary and lymph nodes. This treatment is related to an excellent 5-year disease specific survival of 100%. In stage III B and IV patients’ 5-year survival rates of 97.1% and 84.8% are reported [2, 7].

The benefit of surgical therapy of liver metastasis is discussed controversially. A recent analysis showed that the type of liver resection (anatomic versus non-anatomic) in patients with M1 disease showed higher rates of recurrence after non-anatomic resection but the type of resection did not affect overall survival [8]. Another analysis of 111 patients found that surgical resection influenced mortality after 5 but not after 10 years [9]. It was the aim of this study to compare survival rates of patients with and without liver metastasis and to investigate whether surgery for hepatic metastasis can improve overall survival.

To our knowledge, this is the first study investigating the reasonability of liver surgery in a big cohort of patients with siNENs over a follow-up period of 20 years.

## Methods

In this study we included all patients who had received surgery for the primary tumor ± surgery for liver metastases because of a siNEN between 1965 and 2016 at the Division of Surgery, Medical University of Vienna.

Clinical and therapeutic details were documented for all patients. All histological specimens were revised and reclassified by one pathologist, including immune-histochemical analysis of the Ki-67 proliferation index. The grading (G1–G3) was performed according to the current guidelines [10].

Follow-up was performed at the outpatient clinic of the department of surgery following the current ENETS recommendations [11].

### Statistics

Loss of long-term follow-up was documented in 6/123 (4.9%) patients. These patients were excluded from survival analysis (Table 2). Furthermore, three patients had received a liver transplant, also these patients were regarded as a separate group and not included in the survival analysis (n of patients included in the survival analysis = 114).

Additional adjuvant medical treatment (e.g. somatostatin-analogues, mTOR-inhibitors, RTK-inhibitors, liver-targeted therapies) was discussed in multidisciplinary tumor board meetings and (if feasible) recommended during follow-up and was performed uniformly in patients of all groups according to progress regarding RECIST criteria or contemporary criteria of the respective era the patient was treated in. Therefore, additional loco-regional treatment after surgery or during surveillance may be regarded the same for all patients and is therefore not discussed in detail.

The survival rates were estimated by Kaplan–Meier analysis for patients with and without liver metastasis and according to the type of treatment of liver metastases (surgical vs. surveillance).

In a sub-analysis the patients with small liver resections (≤ 1 segment, group 2a, 15 patients) were merged into one cohort with patients with liver resections of more than one segment (> 1 segment, group 2b, 7 patients). Details of the subgroups are presented in Table 3.

Survival rates of groups were compared using log-rank tests for the overall survival and cox regression was performed. Statistical significance was considered with a *p* < 0.05.

All calculations were done with SPSS Statistics 26.0 and Microsoft Excel 16 for Windows.

## Results

One hundred and twenty-three patients with siNENs of the of the jejunum (n = 22 [17.9%]) or ileum (n = 101 [82.1%]) were treated at the Division of Surgery, Medical University of Vienna within 50 years (1965 and 2016). Seventy one were male (57.7%) and 52 (42.3%) were female (ratio: male:female = 1.37:1); the mean age was 62 (range: 36—87 years).

The primary tumors were classified G1 in 94/123 (76.4%) or low G2 (Ki-67 ≤ 5%) in 29/123 (23.6%). No G3 tumors were verified. Multifocality was seen in 40/123 (32.5%) patients.

In 81/117 (69.2%) liver metastasis were documented. The distribution of the proliferation index was equal and therefore comparable between patients with and without liver metastases.

The detailed staging of the patients is summarized in Table1.

**Table 1:** Demographics

		Total n = 123/117^a^
Gender	Male	71 (57.7%)
	Female	52 (42.3%)
Localization	Jejunum	22 (17.9%)
	Ileum	101 (82.1%)
Grading	G1	94 (76.4%)
	G2	29 (23.6%)
Primary tumors	Solitary	83 (67.5%)
	Multiple	40 (32.5%)
Age (range)		62 (36; 87)
Stage I–III A	N0, M0	35/117^a^ (29.9%)
Stage III B	N1, M0	1/117^a^ (0.9%)
Stage IV	N0/N1, M1	81/117^a^ (69.2%)
	No liver surgery (surveillance-Group 1)	53/81 (65.4%)
	Liver resection ≤ 1 segment (Group 2a)	17/81 (21.0%)
	Liver resection > 1 segment (Group 2b)	8/81 (9.9%)
	Liver transplantation	3/81 (3.7%)^b^

^a^6 patients were lost in long-term follow-up

^b^Excluded from survival analysis

### Overall survival of M0 and M1 patients

**Fig. 1:**
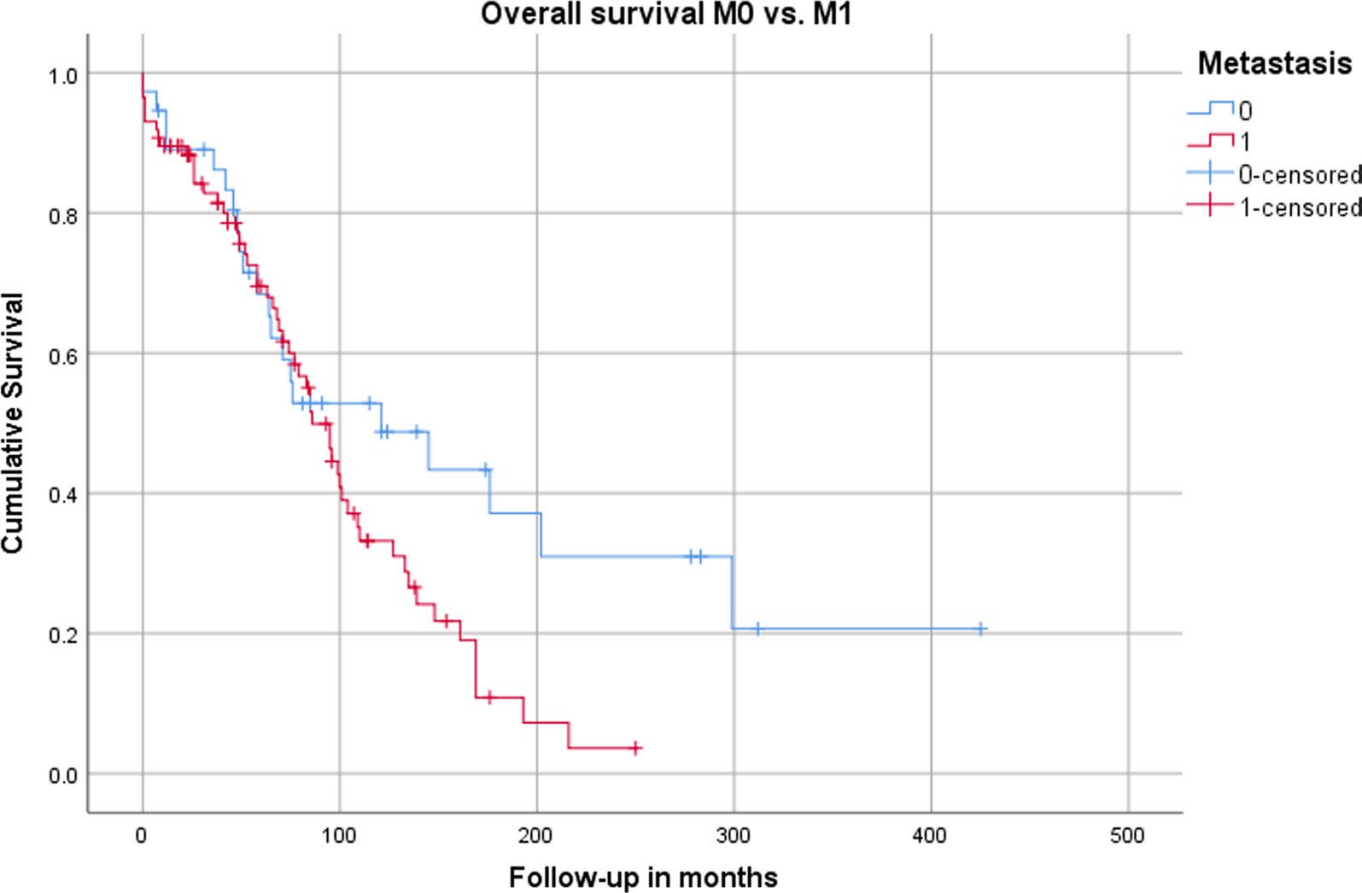
Estimated cumulative survival comparing patients without (0) and with (1) liver metastases

The mean follow-up was 121 ± 49.4 in group M0 and 86 ± 7.6 months in group M1. The overall survival calculation by Kaplan–Meier showed an estimated survival of 176.3 ± 30.3 months for patients without liver metastases and 98.1 ± 8.1 months for patients with liver metastases (Fig. 1). Although there was a clear trend for better survival in group M0 in the overall comparison with the log rank test, the result marginally did not reach statistical significance (*p* = 0.051). The cumulative 1-, 5-, 10- and 20-year survivals calculated by Kaplan–Meier are summarized in Table 2.

**Table 2:** Overall 1-, 5-, 10- and 20-year survival; [patients at risk]

Metastasis		Estimated cumulative survival (Kaplan–Meier)			
		1-year	5-year	10-year	20-year
M0	n = 36/117 (30.8%)	89.0 ± 5.2% [32]	68.4 ± 7.9% [22]	52.8 ± 8.7% [17]	31.0 ± 10.0% [5]
M1	n = 81/117 (69.2%)	89.5 ± 3.3% [76]	69.5 ± 5.4% [46]	33.2 ± 6.1% [17]	3.6 ± 3.4% [1]

### Treatment specific overall survival of M1 patients

**Fig. 2:**
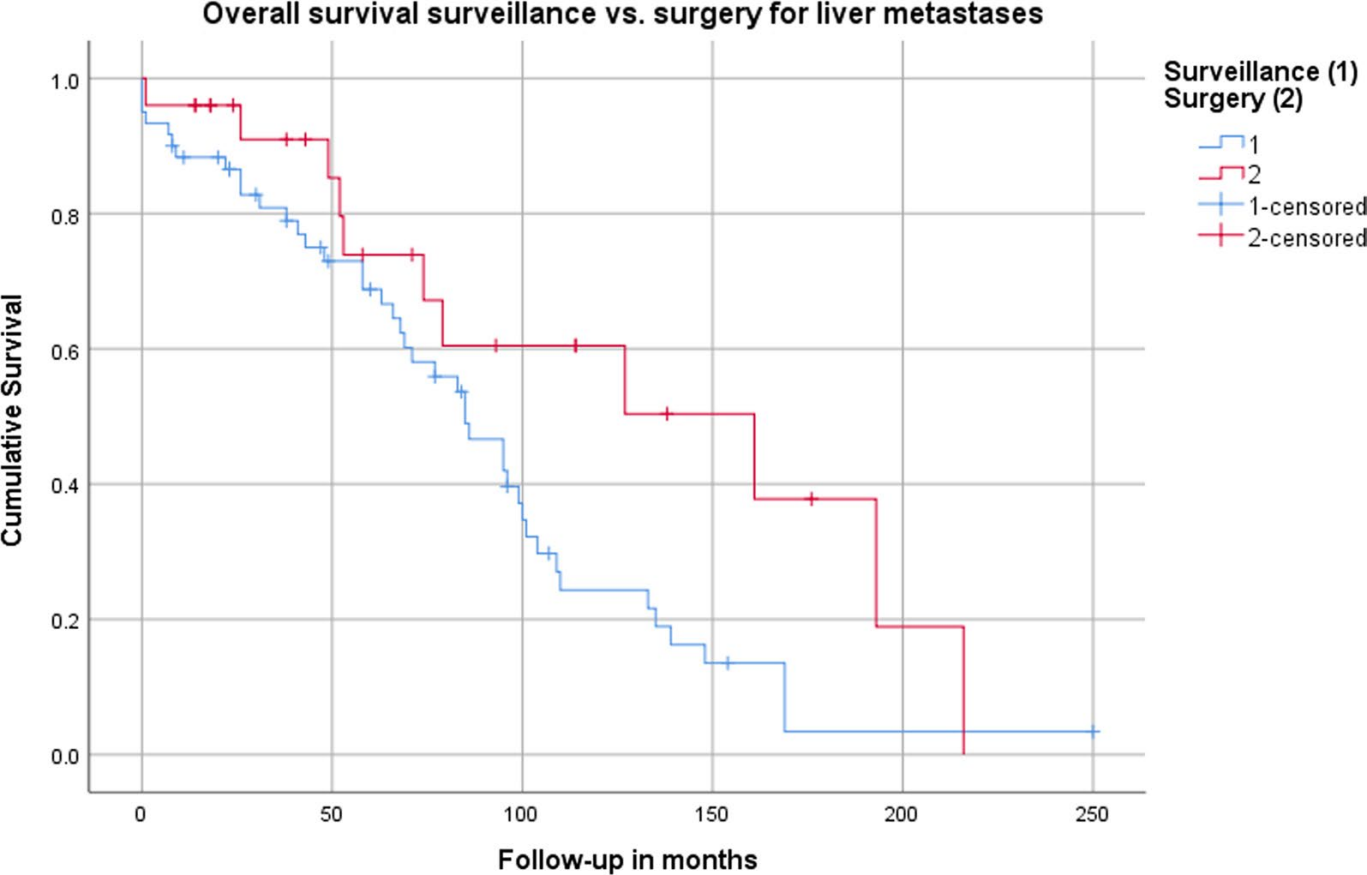
Estimated cumulative survival comparing patients without (1) and with surgery for liver metastases (2)

In the sub-analysis the mean overall survival estimated by Kaplan–Meier for patients without surgery for liver-metastases was 88.1 ± 8.3 months and 130.7 ± 18.5 months for patients who had received liver surgery (Fig. 2). The log rank test shows statistical significance (*p* = 0.04).

The Kaplan–Meier calculation demonstrated a 1-year survival of 86.5 ± 4.4% (patients at risk: 49) for patients without and 96.0 ± 3.9% (patients at risk: 21) for patients with liver surgery.

The 5-year and 10-year survival was 68.8 ± 6.4% (patients at risk: 33) and 24.3 ± 6.6% (patients at risk: 9) for patients with surveillance, 73.9 ± 10.2% (patients at risk: 12) and 60.5 ± 12% (patients at risk: 6) for patients with liver surgery, respectively. The 20-year survival calculations showed that one patient in the group without surgery was still alive. Details, including the sub-analysis according to the extent of surgery are listed in Table 3.

**Table 3:** Overall 1-, 5-, 10- and 20-year survival regarding treatment of liver metastases; [patients at risk]

Treatment			Estimated cumulative survival (Kaplan–Meier)			
			1-year survival	5-year survival	10-year survival	20-year survival
Group 1	Surveillance	n = 52	86.5 ± 4.4% [49]	68.8 ± 6.4% [33	24.3 ± 6.6% [9]	3.4 ± 3.2 [1]
Group 2	Surgery	n = 22	96.0 ± 3.9% [21]	73.9 ± 10.2% [11]	60.5 ± 12% [6]	0
Subgroup 2a	Minor liver surgery (≤ 1 segment)	n = 15	94.1 ± 5.7% [14]	76.7 ± 12.2% [6]	63.9 ± 15.5% [3]	0
Subgroup 2b	Major liver surgery (> 1 segment)	n = 7	100% [7^a^]	71.4 ± 17.1% [5]	57.1 ± 18.7% [3]	0

^a^Only one patient remained free of liver-metastases during follow-up

No statistical significance with regard to overall survival was documented comparing group 2a (minor liver surgery) and 2b (major liver surgery).

### Multivariate analysis

A cox proportional hazards regression analysis was performed on individual risk factors (presence of liver metastases; grading: G1 vs. G2; multifocality; surgery vs. surveillance) and showed that only the presence of liver metastases at diagnosis was an individual risk factor for worse survival (hazard ratio: 2.371).

## Discussion

Due to a long indolent course, many patients with siNENs are diagnosed in a late, metastasized state. Therefore, not surprisingly, distant metastases were found in 81/117 (69.2%) patients, which is a higher number than reported in a former analysis [6]. Current literature controversially discusses the surgical treatment of (asymptomatic) primary tumor(s) and lymph node metastasis and the influence on prognosis in patients with verified liver metastases [12–15]. However, primary tumors and lymph node metastases may cause bowel obstruction or obstruction of the blood supply of the intestine resulting in life threatening ischemic damage of the intestine [2]. Therefore, all patients who are included in the current analysis had surgery of the primary tumors and of affected lymph nodes.

Medical treatment with somatostatin analogues is state of the art in stage IV patients [16, 17]. However the surgical treatment of liver metastases is still an ongoing matter of debate [18, 19]. Debulking liver surgery may be indicated to reduce serotonin-producing tumor mass in patients who are symptomatic because of hormone excess leading to diarrhea with electrolyte deficiency, flushing, sweating, palpitations or Hedinger’s syndrome. In current guidelines detailed information is published on systemic therapy [18]. However recommendations for surgery in asymptomatic or minimally symptomatic patients with liver metastasis are lacking [20, 21]. Recently a benefit of surgery of liver metastasis of neuroendocrine neoplasia of the pancreas was shown in regard to overall survival, however there is little data on siNENs [22]. Moreover, patients with siNENs early demonstrate multiple (in the majority small) bilobar liver metastases and curative surgery is rarely possible [19].

In the current patient cohort patients verified with liver metastasis have a similar 1- and 5-year overall survival compared to patients with surgically treated regionalized disease (primary tumor removed and lymph node metastases dissected). This may be explained by the slow growth of liver lesions and the good interaction of generalized medical treatment and/or liver-targeted therapies [18, 23, 24]. The estimation of the mean overall survival of patients without liver metastases (176.3 ± 30.3 months) compared to patients with liver metastases (98.1 ± 8.1) shows a clear trend in favor of patients with regionalized disease, however statistical significance was not reached (*p* = 0.051). Comparing the 10-year overall survival of M0 (52.8 ± 8.7%) and M1 patients (33.2 ± 6.1%) a clear trend was documented and multivariate analysis showed that the presence of liver metastases was an individual risk factor associated with worse overall survival in our patient cohort. Interpretation of the results has to be done with caution because the low number of patients (17 patients at risk in each group) is a severe limitation of the study. Interestingly, the analysis showed that patients with liver metastases hardly ever survived for 20 years compared to 31.0% of patients without liver metastases.

The sub-analysis comparing overall survival of “surveillance” and “surgery” of liver-metastasis showed a statistically significant impact in favor of surgery (no surgery: 88.1 ± 8.3 months vs. surgery: 130.7 ± 18.5 months; *p* = 0.04). Interestingly, the short-term overall survival of up to 5-years shows equal results between the two groups (no surgery: 68.8 ± 6.4% vs. surgery: 73.9 ± 10.2%). This finding underlines the importance of long-term treatment options in patients with siNENs; because of their very slow growth differences between therapeutic approaches may not be seen before 10-years of follow-up (no surgery: 24.3 ± 6.6% vs. surgery: 60.5 ± 12%). In almost the same manner the conclusion has to be drawn with caution because of a very low number of patients in the 10 year follow up (no surgery: 9, surgery: 6). It needs to be emphasized that this analysis is retrospective and that the treatment plan for each patient was made individually taking into consideration multiple factors that influence the decision whether the patient is a candidate for liver surgery. Therefore, multiple factors must be regarded as a possible bias, i.e. comorbidities that make the patient not fit for liver surgery.

Concerning the extent of liver surgery, no differences in survival were seen between patients with resection of “up to one” or “more liver segments”. This may be explained by the extent of liver-disease at the time of surgery which is in the majority of patients more extended than documented by preoperative functional staging. The long term survival of the patients analyzed after 10-years is in concordance with literature [9].

## Conclusions

SiNENs are slowly growing tumors, even in patients with liver metastases the overall survival is very good and not significantly worse compared to patients without liver metastases. However, those patients who received surgery for liver metastases have a better outcome compared to patients whose liver metastases are not removed surgically. This result needs to be interpreted with caution because only patients who are fit for surgery and have metastases that can be sensibly treated by surgery did receive this treatment. Therefore, feasibility to perform surgery on liver metastases in patients with siNENs should be considered especially in patients symptomatic due to high tumor burden and serotonin hypersecretion to reduce hormone activity.

## Data Availability

All data and material analyzed in this study is available.

## Abbreviations

5-HT: Serotonin
ENETS: European neuroendocrine tumor society
Ki-67: Kiel-67
mTOR: Mechanistic target of rapamycin
NEN: Neuroendocrine neoplasia
NET: Neuroendocrine tumor
RTK: Receptor tyrosine kinase
siNEN: Small intestinal neuroendocrine neoplasia
